# Tackling potentially inappropriate prescriptions in older adults: development of deprescribing criteria by consensus from experts in Colombia, Argentina, and Spain

**DOI:** 10.1186/s12877-023-04271-9

**Published:** 2023-10-20

**Authors:** Roxana De las salas, Claudia Vaca-González, Javier Eslava-Schmalbach, Catalina Torres-Espinosa, Albert Figueras

**Affiliations:** 1 Department of Nursing, Km5 Via Puerto Colombia, Universidad del Norte, Barranquilla, Colombia; 2 Faculty of Science, Department of Pharmacy, Universidad Nacional de Colombia, Carrera 45 N° 26-85, Bogota, Colombia; 3 Faculty of Medicine, Department of Surgery, Universidad Nacional de Colombia, Carrera 45 N° 26-85, Bogota, Colombia; 4 Faculty of Medicine, Department of Internal Medicine, Universidad Nacional de Colombia, Carrera 45 N° 26-85, Bogota, Colombia; 5 Faculty of Medicine, Autonomus University of Barcelona, Bellaterra (Cerdanyola del Vallès), 08193 Barcelona, Spain

**Keywords:** Deprescriptions, Polypharmacy, Inappropriate Prescribing, Geriatrics, Potentially inappropriate medication

## Abstract

**Background:**

Potentially inappropriate medication use is prevalent among older adults in primary care, leading to increased morbidity, adverse drug reactions, hospitalizations, and mortality. This study aimed to develop and validate a tool for identifying PIMs in older adults within the primary care setting. The tool is composed of a list of criteria and was created based on consensus among experts from three Spanish-speaking countries, including two from Latin America.

**Methods:**

A literature review was conducted to identify existing tools, and prescription patterns were evaluated in a cohort of 36,111 older adults. An electronic Delphi method, consisting of two rounds, was used to reach a formal expert consensus. The panel included 18 experts from Spain, Colombia, and Argentina. The content validity index, validity of each content item, and Kappa Fleiss statistical measure were used to establish reliability.

**Results:**

Round one did not yield a consensus, but a definitive consensus was reached in round two. The resulting tool consisted of a list of 5 general recommendations per disease, along with 33 criteria related to potential problems, recommendations, and alternative therapeutic options. The overall content validity of the tool was 0.87, with a Kappa value of 0.69 (95% CI 0.64—0.73; Substantial).

**Conclusions:**

The developed criteria provide a novel list that allows for a comprehensive approach to pharmacotherapy in older adults, intending to reduce inappropriate medication use, ineffective treatments, prophylactic therapies, and treatments with an unfavorable risk–benefit ratio for the given condition. Further studies are necessary to evaluate the impact of these criteria on health outcomes.

**Supplementary Information:**

The online version contains supplementary material available at 10.1186/s12877-023-04271-9.

## Introduction

Potentially inappropriate medication (PIM) refers to the use of medications that pose a higher risk than their potential benefit, particularly when safer alternatives exist for treating the same condition [1]. PIMs are prevalent among older adults and can contribute to various negative outcomes, including increased morbidity, prescription errors, therapeutic duplications, unnecessary medication, incorrect dosing, and prolonged prescriptions, ultimately leading to adverse drug reactions, hospitalizations, and mortality [2].

Explicit and implicit tools are commonly used to measure PIMs. Explicit tools rely on predefined criteria, while implicit tools involve professional judgments. Among the most widely recognized tools for identifying PIMs are the STOPP/START criteria (Screening Tool of Older People's Prescriptions/Screening Tool to Alert to Right Treatment) [3] and the AGS (American Geriatric Society) Beers criteria [4]. These tools consist of evidence-based recommendations specific to medication use in older adults.

The original Beers Criteria were developed in 1991 based on findings from an American prescription data set. A team of experts from the United States and Canada [5] subsequently reached a consensus on explicit criteria defining inappropriate medication use in a nursing home population. Similarly, the initial version of the STOPP/START criteria was developed through a comparable process involving experts from Ireland and the United Kingdom [6]. While these evidence-based criteria are well-validated and valuable, they do not encompass prescribing patterns in low- and middle-income countries (LMICs), which may differ from those in high-income countries (HICs) due to factors such as pharmaceutical market composition, cultural influences, educational factors, marketing exposure, and other determinants of physicians' prescribing habits.

Although the practice of deprescribing in older adults has gained support through various criteria [1, 5–9], existing tools often include medications that are not commonly used in LMICs and fail to consider epidemiological differences between HICs and LMICs. Additionally, specific criteria for newly introduced medicinal products, which are increasingly utilized, remain scarce [10]. As a result, new lists of PIMs adapted to specific contexts have emerged [11–13].

Several studies have demonstrated the success of deprescribing across numerous pharmacological groups [14–16], supported by criteria, tools, and concepts. However, there is a lack of guidelines and criteria for discontinuing medications [17, 18] in comparison to the abundance of guidelines for prescribing. Hence, the objective of this study was to develop and validate a list of deprescribing criteria for inappropriate medications in older adults. This was achieved by considering prescription patterns in a cohort of 36,111 older individuals with chronic diseases in Colombia and obtaining expert consensus from Latin America and Spain. The focus was on prescriptions commonly associated with health conditions prevalent in this age group.

The methodology employed in this study follows a similar process to that used in the development of the STOPP/START and Beers criteria, as discussed previously.

## Methods

**Fig. 1:**
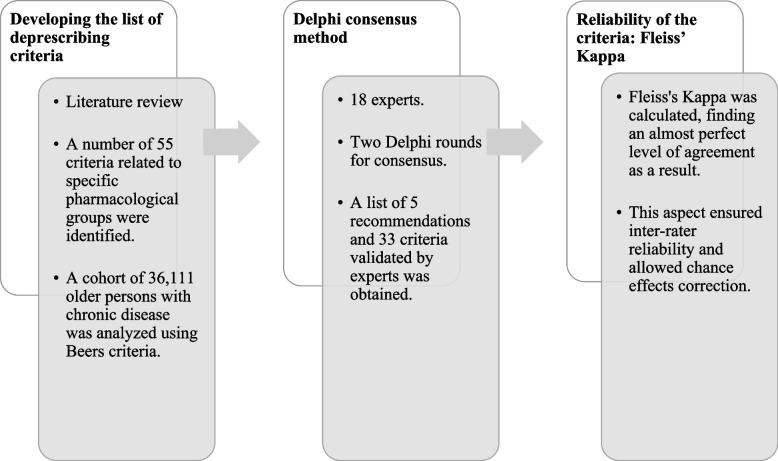
Steps of the development of deprescribing criteria

To define the deprescribing criteria included in the list and establish their validity, three consecutive phases were implemented, as depicted in Fig. 1.

### Phase 1: Developing the list of deprescribing criteria

The list was constructed through a systematic literature review [19] and an analysis of prescription patterns in a cohort of 36,111 older adults (≥ 60 years) enrolled in the Colombian healthcare system. The literature review helped identify existing tools and assess the relevance of therapeutic groups commonly used in older populations. The analysis of prescription patterns in the cohort enabled the identification of gaps in criteria for evaluating inappropriate medication. These sources of information were used to propose criteria, considering the following aspects:

1. Chronic medication with potentially inappropriate use (excluding treatments for acute conditions).
2. High-consumption medications with potentially inappropriate use.
3. Medications with low therapeutic value (demonstrating low or no efficacy in older adults).
4. Prophylactic treatments.
5. Treatments with an unfavorable risk–benefit ratio considering factors such as age, pharmacokinetic changes, and life expectancy.

### Phase 2: Delphi consensus method

#### Preconsensus

A preliminary consensus was reached among three clinical pharmacologists and two family medicine physicians. A total of 40 pharmacotherapeutic groups (consisting of 60 different active pharmaceutical ingredients), five recommendations, and 61 potential problems to evaluate were submitted for review.

#### Rounds

A panel of 20 experts from diverse professions and specialties were invited to participate in the formal consensus using an electronic Delphi method. Eligibility criteria used for experts’ selection were at least 5 years of experience in the field (clinical, academy, or research), at least a medical specialty, master’ or doctoral degree. Of the 20 experts invited, 18 agreed to participate, including 5 geriatricians, 2 internal medicine physicians, 1 endocrinologist, 3 general physicians, 2 pharmacologists, 3 clinical pharmacists, 1 family medicine physician, and 1 nurse. Two rounds were conducted to achieve consensus and establish the face and content validity of the proposed criteria.

In the first round, 16 experts participated and were given two weeks to provide their level of agreement regarding the inclusion of each criterion. The second round involved 18 experts and aimed to address any disagreements that arose during the first round. Participants were again given two weeks to submit their responses. The second round resulted in a definitive consensus. Feedback on the results was provided in both rounds using descriptive measurements such as medians and ranges.

#### Phase 3: Validity and reliability of the criteria

Validity and reliability are crucial in assessing the consistency, stability, and replicability of expert opinions regarding the proposed criteria. These factors are essential for establishing trust and facilitating the use of criteria by other researchers and physicians. In this study, reliability was evaluated using Fleiss' Kappa statistic, and content validity (RCV) was assessed using Lawshe's scale modified by Tristan [20]. Additionally, the global reliability of the tool was determined using the validity content index.

Interevaluator reliability was evaluated by measuring the level of agreement among experts on a Likert scale ranging from 1 to 9 (1 = completely disagree, 9 = completely agree). The tool demonstrated acceptable levels of precision, clarity, and understandability, with a minimum value of 0.7 (substantial agreement), as determined by Fleiss' Kappa statistic:

$$\underline{K} =1 - \frac{n {m}^{2} - {\sum }_{i=1}^{n}{\sum }_{j=1}^{r}{x}_{ij}^{2}}{n m \left(m-1\right){\sum }_{j=1}^{r}{\underline{p}}_{j} {\underline{q}}_{j}}$$

where n is the total number of categories, m identifies the number of raters, xij defines the number of registers of category i in category j, r indicates the number of categories that constitute the nominal system, p indicates the proportion of positive agreements among raters, and q indicates the proportion of negative agreements among raters (1 – p).

The rate of content validity (RCV) for each item was evaluated using a three-degree range scale (not necessary, useful but not essential, and essential). The minimum acceptance index, according to Lawshe modified by Tristan [20], was set at 0.58.

### Statistical analysis

The level of agreement among experts was reported using medians and ranges. Interevaluator reliability was assessed using the Kappa Fleiss statistic, and the 95% confidence interval (CI) was calculated using the R program version 3.6.0.

### Ethics declarations

The study protocol received approval from the Ethics Committee of the Facultad de Ciencias (Science Faculty) of the Universidad Nacional de Colombia (National University of Colombia) (ID: 06–2017). Experts were asked to provide their consent to participate in the two-round Delphi method.

## Results

### Phase 1: Development of the deprescribing criteria list

The deprescribing criteria list was developed based on 55 published tools identified in the literature [19] and a descriptive observational study conducted on a retrospective cohort of 36,111 outpatient older adults with chronic illnesses (see Additional file 1). These individuals were receiving care in primary healthcare facilities from January to June 2017 and were affiliated with the healthcare system. Electronic records were obtained from a nationwide pharmacy service, which serves approximately 14 million patients across the country.

The study cohort had a minimum age of 60.08 years and a maximum age of 106.08 years, with a median age of 70.41 years (SD: 7.89) (see Additional file 2: Sociodemographic and clinical characteristics of the cohort). Using the Anatomical, Therapeutic, Chemical (ATC) classification system, the most commonly prescribed medications were those used in diabetes, which accounted for 90% of the cases. They were followed by agents acting on the renin-angiotensin system (66.86%), lipid modifying agents (66.78%), and antithrombotic agents (55.95%), among others. Among the drugs used in diabetes, the most frequently prescribed were biguanides (metformin) in 48.38% of the cases, sulfonylureas in 12.70%, fast-acting insulins and analogs in 6.93%, and long-acting insulin analogs in 16.47%. Notably, a therapeutic shift toward long-acting insulin analogs was observed (see Additional file 3: Pharmacological groups prescribed).

In this study, at least one PIM was identified in 23.39% of the older adults. The most commonly prescribed medications classified as PIMs were proton pump inhibitors (23.39%), followed by sulfonylureas (13.67%), antidepressants (7.58%), and NSAIDs (3.45%). Additionally, other therapeutic groups of interest were identified, including antidiabetic medications (insulins, oral antidiabetics), statins, and bisphosphonates, which are not found in other criteria. These findings allowed us both to prioritize certain criteria related to these pharmacological groups and their associated substances and to suggest recommendations for reorienting or reassessing therapeutic objectives. Afterward, the proposed criteria were subjected to expert preconsensus.

### Phase 2: delphi consensus method

#### Round 1

During the first round, the median values (x̃) for the criteria and recommendations ranged from 7 to 9. However, consensus was only reached for recommendation 6 and criteria 12, 13, 17, 20, 26, 30, and 32 (agreement level between 7 and 9). Recommendations 1, 3, 4, and 6, as well as criteria 3, 8, 14, 16, 19, 22, and 28, achieved relative consensus (agreement level between 4 and 9). The remaining criteria and recommendations did not reach a consensus during this round.

Recommendations provided by the experts were received, and as a result, criterion 20 was divided into two separate criteria: one related to theophylline and another related to inhaled corticosteroids. Additionally, a criterion on laxatives was recommended and subsequently accepted and included in the second round. Since no consensus was reached, a new round was deemed necessary.

#### Round 2

During the second round, the median values ranged from 7 to 9. Since the agreement levels on the Likert scale ranged from 7 to 9, definitive consensus was reached during round two regarding the proposed criteria and recommendations (Additional file 4).

As a result, a final list was compiled, consisting of five general recommendations for reassessing therapeutic objectives in type 2 diabetes mellitus (DM2), arterial hypertension, dyslipidemias, and disorders of the central nervous system. Additionally, the list included thirty-three criteria related to different pharmacological groups and/or active pharmaceutical ingredients. Each criterion was accompanied by the associated potential problem to evaluate, recommendations, and alternative therapeutic options (Additional file 1).

#### Phase 3: validity and reliability of the criteria

During round two, the content validity was assessed. The recommendations and criteria demonstrated a content validity ratio (CVR) above 0.58, indicating their acceptance. However, criterion 34 concerning laxatives had a CVR of 0.38, leading to its exclusion from the final list. The CVR for the accepted criteria ranged from 0.77 to 1.00. The overall content validity index for the accepted criteria was 0.87.

The general Kappa Fleiss value for the accepted criteria was 0.69 (95% CI 0.64–0.73). According to the strength of agreement, the reliability between evaluators ranged from moderate to almost perfect (see Additional file 4). These results confirm that the proposed criteria represent a validated list of pharmacological groups.

## Discussion

This is the first criteria list developed by experts from LMICs to identify PIMs, taking into consideration published criteria from around the world. The proposed deprescription criteria provide a critical evaluation of the clinical evidence regarding medications that should be avoided in older adults and the need to improve their usage. The final list comprises 5 recommendations aimed at re-evaluating therapeutic objectives in health conditions such as type 2 diabetes mellitus, hypertension, dyslipidemias, and disorders of the central nervous system. These conditions were found to be associated with the highest occurrence of PIMs in the analyzed cohort and are underrepresented in existing criteria sets such as STOPP/START [3] and BEERS [4]. The clinical evidence was thoroughly reviewed during the preconsensus round as well as in the two rounds of expert consensus (Additional file 1).

After the process, a total of 33 criteria related to pharmacological groups (corresponding to 49 substances) were included, along with 63 potential problems to evaluate. Additionally, recommendations and alternative therapeutic options were incorporated concerning the proposed deprescription criteria.

Among these 33 criteria, 23 are related to the STOPP/START criteria [3], while 20 are related to the Beers criteria [4]. The remaining 10 criteria are novel and address the treatment of type 2 diabetes mellitus, including insulin (alone or in combination with liraglutide), glargine insulin, GLP-1, IDPP-4, and ISGLT-2 inhibitors. Other criteria are relevant to chronic obstructive pulmonary disease (inhaled corticosteroids), osteoarthritis (diacerein, glucosamine, chondroitin), and various cardiovascular conditions (fibrates and potassium-sparing diuretics). These criteria fill the gaps in existing tools and cover medications commonly used by older adults.

Previous studies by Qaseem et al. [21] have established treatment objectives of A1c hemoglobin for glycemic control in adults with pharmacological therapy. Similarly, Mallery et al. [22] developed criteria based on evidence for treating frail older adults with type 2 diabetes. In this last group, the criteria to reduce excessive medication in diabetes are even clearer.

Regarding antihypertensive medications, Lavan et al. [23] highlighted the likelihood of alpha-blockers causing orthostatic hypotension and falls in older adults, making their discontinuation widely accepted. Likewise, Mallery et al. proposed evidence-based criteria for treating frail older adults with arterial hypertension [24] and with dyslipidemias [25]. In all these cases, the criteria for reducing excessive medication are well defined, particularly in the presence of frailty, reduced life expectancy, or when risks outweigh potential benefits. However, it is important to emphasize that the criteria proposed by Lavan et al. [23] and Mallery et al. [24, 25] focus exclusively on frail adults and do not encompass outpatient older adults with chronic conditions, which is the target population for our criteria in the primary care setting.

The development of country-specific lists of potentially inappropriate medications is a growing trend and essential for enhancing the quality and safety of pharmacotherapy in daily practice. In a prospective study, Hamilton et al. [26] found that the STOPP criteria in hospitalized older adults were predominantly associated with adverse drug reactions (ADRs), unlike the Beers criteria. Budnitz et al. [27] reported that the majority of adverse reactions were caused by the use of antiplatelet medication, anticoagulants (warfarin), insulins, and oral hypoglycemic agents. These medications are included in our proposed list of criteria.

Our present proposal introduces five new criteria for insulin degludec (alone or in combination with liraglutide), insulin glargine, GLP-1 (glucagon-like peptide 1 receptor agonists), DPP-4 inhibitors (inhibitors of dipeptidyl peptidase 4), and SGLT-2 inhibitors (sodium-glucose cotransporter-2 inhibitors). These medications were not included in the 2015 STOPP version [3] or the AGS Beers 2019 [4], but they better align with our specific context and medication availability.

AGS Beers 2019 [4], Renom-Guiteras et al. [28], and Pruskowski et al. [29] include short- and long-acting insulins but do not specify long-acting insulin analogs. Kojima et al. [12], Farrell et al. [30], and PHN Tasmania (Primary Health) [31] refer to the risk of severe hypoglycemia associated with all insulins. Mallery et al. [22] recommend the use of NPH insulin (Neutral Protamine Hagedorn) in patients nearing the end of their lives, avoiding the use of long-acting insulin analogs such as insulin glargine or insulin detemir, as they do not appear to provide significant clinical benefits compared to insulin NPH and are also more expensive [32, 33]. These considerations are crucial in LMICs due to medication accessibility issues.

On the other hand, FIMEA [34] and MedStopper [35] provide extensive references to insulins, including analogs. Our inclusion of insulin analogs reflects changes in prescription patterns observed in older adults, emphasizing the higher risk of severe hypoglycemia and the complexity of managing them in this population [32].

Our proposal also includes GLP-1 analogs, which are only minimally considered in other proposals such as the FORTA (Fit fOR The Aged) list [36], PHN Tasmania [31], MedStopper [35], and Mallery et al. [22]. While DPP-4 inhibitors are included in the FORTA [36], FIMEA [34], PHN Tasmania [31], Renom-Guiteras et al. [28], Pruskowski et al. [29], and Mallery et al. [22] lists, SGLT-2 inhibitors are only listed in PHN Tasmania [31].

A systematic literature review revealed that antihyperglycemic medications can be safely deprescribed in older adults [37]. Similarly, the implementation of deprescribing strategies within the context of clinical research has demonstrated their safety [15, 18].

Our proposal not only includes the description of potential medications for deprescription but also provides recommendations and therapeutic alternatives. While the STOPP criteria [4] do not incorporate elements of clinical relevance or severity, the AGS Beers criteria focus on deprescribing recommendations based on levels of evidence. In any case, the explicit criteria aim to improve appropriate medication use and prevent potentially serious adverse drug reactions. Both tools have demonstrated their relevance in reducing rates of adverse drug reactions or events when prospectively applied in older adults within specific clinical environments [38, 39].

The Delphi panel consensus methodology was employed to select PIM criteria and define prevailing alternative treatment options when deemed relevant. Sixteen experts participated in the first round, and eighteen experts participated in the second round. Similar rounds of evidence review, language clarifications, and justifications were conducted in the updates of the American Geriatrics Society (AGS), Beers [4], and STOPP [3] criteria through consensus methods. The 2019 version of the Beers criteria involved thirteen experts, and the version 3 of STOPP criteria (screening tool of older people's prescriptions) included a panel of experts [40].

Using a methodology similar to the one described by Ramdomski [41], relevant therapeutic groups and medications in our context were prioritized to construct new deprescribing criteria aligned with those priorities. The results of this exercise were validated through the Delphi methodology with the participation of experts in geriatrics, family medicine, internal medicine, endocrinology, pharmacology, clinical pharmacy, and nursing. Based on this methodology, a deprescribing algorithm was designed [42], accompanied by four recommendations and 33 criteria to support medication deprescription.

The main limitation of our criteria list is the lack of a study validating its use in ambulatory and/or clinical environments for older adults. However, a robust process was undertaken to assess face and content validity, similar to other tools [28, 36, 42]. This means that our criteria list includes recommendations, substances and/or pharmacological groups, and the primary reasons why deprescription should be considered. This is based on both evidence and the opinions of the participating experts. Additionally, validation is an ongoing process that is reinforced through the use of the tools. Although there is mounting evidence of the benefits, evaluating the impact of such tools is necessary for their adoption in public health policies.

Another potential limitation is the Delphi methodology, which manages ideas using a questionnaire without open discussion. However, this is mitigated by the involvement of a small expert group. On the other hand, the Delphi method offers several advantages over other research methodologies when addressing research problems or questions with no clear or no existing data-driven answer.

Finally, we acknowledge that the newly proposed criteria are limited to a prescription cohort from a single Latin American country and thus may not fully represent trends in other countries, despite similarities among them. Nevertheless, this is the first tool developed using a data set from an LMIC, and the medication used by this cohort better reflects the regional pharmaceutical market compared to other widely used tools. Additionally, the expert group comprising professionals from various Latin American countries and Spain understands prescription behaviors and the clinical evidence supporting their decisions. The process of proposing the criteria and reaching a consensus was similar to that of other recognized tools, and it should be periodically reviewed and updated to capture changes in prescription patterns and the availability of newer medications for older patients.

## Conclusions

The formulation of five recommendations and 33 criteria represents a prioritized approach to addressing the pharmacotherapy needs of older adults, intending to minimize inappropriate medication use. These validated criteria offer practical evidence-based recommendations to guide decision-making regarding the prescription of chronic medications, with a particular focus on assessing their potential benefits within the context of patient life expectancy and care objectives in primary care settings.

The established criteria enable the evaluation of various medication categories, including chronic medications, high-consumption medications, and medications with limited or no efficacy in older adults. They also consider prophylactic treatments with an unfavorable risk–benefit ratio for specific health conditions. These potentially inappropriate medications were identified through a prescription data set and a consensus process involving participants from three Latin American countries and Spain. This cohort better represents the regional pharmaceutical market compared to other widely used tools.

The deprescribing criteria demonstrate high rates of agreement, validity, and relevance, as evidenced by their development through the Delphi consensus method, which aligns with other research studies. It is important to note that the proposed tool must undergo periodic reviews and updates to adapt to changes in prescription patterns and the availability of newer medications for the treatment of older patients. This dynamic process ensures the continued relevance and effectiveness of the criteria.

### Supplementary Information

**Additional file 1.** Deprescribing criteria for potentially inappropriate medication in older persons.

**Additional file 2.** Sociodemographic and clinical characteristics of the cohort (*n*=36111).

**Additional file 3.** Pharmacological groups prescribed.

**Additional file 4.** Validity and Reliability of some criteria.

## Data Availability

All data generated or analyzed during this study are included in this published article and its supporting information files (dx.doi.org/10.17504/protocols.io.bp2l6x2bklqe/v1); further inquiries can be directed to the corresponding author/s.
