# Supplementary material for: Tackling potentially inappropriate prescriptions in older adults: development of deprescribing criteria by consensus from experts in Colombia, Argentina, and Spain
Source: BMC Geriatr. 2023 Oct 20;23:682. doi: 10.1186/s12877-023-04271-9 (PMC10588094; doi:10.1186/s12877-023-04271-9)
Supplement: Supplementary file 1 — Additional file 1. Deprescribing criteria for potentially inappropriate medication in older persons. [file 12877_2023_4271_MOESM1_ESM.docx]

**Additional file 1. Deprescribing criteria for potentially inappropriate medication in older persons.**

**Final proposal.**

The criteria proposed are built from recommendations that allow the evaluation of therapeutic objectives. A health condition is established, next to a criterion, its potential problem or associated risk, recommendations and alternatives. **R: Recommendation. C: Criterion.**

| **Health Condition** | **Recommendation** | **Reference** |
| --- | --- | --- |
| **R-1. Type 2 Diabetes Mellitus (T2DM)** | *Recommendation.* Avoid aiming for an HbA1c serum level in the presence of:   - A life expectancy < 10 years due to old age (≥ 80 years old), when the patient is institutionalized in a residence and/or has a severe chronic health condition (dementia, cancer, severe chronic kidney disease, severe chronic obstructive pulmonary disease, end organ damage, deterioration of cognitive and sensory status, heart function or congestive heart failure), and poor social/family support, as the potential damages outweigh the potential benefits. Consider that an HbA1c level of 8.5% and a fasting glucose level of 200mg/dL can be acceptable values. A fasting glucose level of 250md/dL at the end of life may be acceptable. - With a good life expectancy (≥10 years), an HbA1c level of 7-7.5% may be appropriate. The therapeutic approach to the disease is multidisciplinary and includes both, pharmacological and non-pharmacological strategies. | Farrell et al, 2017 (1)  Qaseem et al., 2018 (2) |
| **R-2. (T2DM)** | - For patients with symptoms of hypoglycemia (tremors, sweating, irritability, hunger, dry mouth, headaches) and/or an HbA1c ≤6.5% and functionality impairment, it is recommended to deintensify (de-escalate) medication. | ICS, 2018 (3) |
| **High blood pressure**  **R-3** | - In hypertension, a systolic blood pressure <120 mm Hg can represent a risk. | JNC 8, 2014 (4) Solini &Grossman, 2016 (5) |
| **R-4. Dyslipidemia** | - It is recommended to start the treatment with a low to moderate dose of statins if there is coronary risk (i.e. >10% REGICOR). They can be used in secondary prevention even in adults ≥75 years old. They are not recommended for primary prevention in patients ≥80 years old. Beware when using long-acting statins as they might increase glycemia. Diet and increasing physical activity (if tolerated) is recommended. | Cui et al, 2018 (6)  Wilson et al, 1998 (7) |
| **R-5. CNS disorders** | - Start the treatment with the lowest doses, evaluate dose reduction and/or intermittent use, use short or intermediate-acting drugs in order to avoid building-up and prolonging of effects. Avoid central acting drug duplicity. Evaluate the presence of drug interactions, risk factors, patient fall risk, history of falls, frailty, motor or cognitive alterations or dementia. Always consider non-pharmacological treatments (walks, music, dancing, etc.). | Fuentes & Mérida, 2011 (8) |

| **Criteria #** | **Drug / Class** | **Associated potential problem/ Reason to evaluate and/or consider suspending** | **Recommendation/ Alternative** | **Reference** |
| --- | --- | --- | --- | --- |
| **C-1** | Insulin degludec  In combination with liraglutide. | Higher risk of prolonged hypoglycemia due to its duration of action.  Higher risk of pancreatitis and/or liver failure.  Consider that the efficacy is limited in patients >65 years old; there is no experience in patients >75 years old. | Avoid in patients with symptoms of hypoglycemia, with low food intake, malnourished, low BMI, sarcopenia; patients with a history or risk of pancreatitis and/or severe liver failure.  Alternative: NPH insulin. | AEMPS, 2018 (9)  Rosenstock y cols. 2018 (10) |
| **C-2** | Insulin glargine | Higher risk of prolonged hypoglycemia due to its duration of action.  Similar efficacy when compared to other less expensive, available options. | Avoid in patients with symptoms of hypoglycemia, with low food intake, malnourished, low BMI, sarcopenia.  Alternative: NPH insulin. | ICS, 2018 (3)  Rosenstock et al. 2018 (10)  Rys et al. 2015 (11)  CADTH, 2010 (12)  ORIGIN Trial (13) |
| **C-3** | *GLP-1 (Glucagon-like peptide 1 receptor agonists)*  (exenatide, liraglutide, lixisenatide, dulaglutide) | May cause rapid weight loss (>1.5 kg per week).  Risk of hypoglycemia in combination with sulfonylureas and/or insulin.  Risk of acute pancreatitis and/or increase of pancreatic lipase and/or pancreatic amylase levels.  Limited efficacy as a monotherapy. Similar efficacy when compared to other less expensive, available options. | Avoid in frail patients with nutritional risk.  Adjust dosing in patients with symptoms of hypoglycemia. Avoid combination during low food intake, malnourishment, low BMI.  Avoid in patients with a history of pancreatitis.  Treatment as monotherapy: not recommended. Evaluate alternatives of choice: metformin and/or sulfonylureas like glimepiride, gliclazide or DDP-4 inhibitors. | AEMPS, 2016 (14)  Marso et al, 2016 (15) |
| **C-4** | *DDP-4 (dipeptidyl peptidase 4 inhibitors) (*sitagliptin, vildagliptin, linagliptin, alogliptin) | Risk of pancreatitis and/or pancreatic cancer. | Avoid in patients with a history or at risk of pancreatitis. Adjust dosing in case of severe renal failure (except linagliptin). Evaluate alternatives: metformin and/or recommended sulfonylureas (glimepiride, gliclazide). | FDA, 2014 (16)  White et al, 2011 (17)  Rosenstock et al, 2019 (18)  Abrahami et al, 2018 (19) |
|  | Saxagliptin | Increased risk of hospitalization in patients with heart failure (HF).  Risk of severe joint pain. | Avoid if there are risk factors of hospitalization due to HF.  Evaluate and/or avoid in patients with arthralgia. |  |
|  | Vildagliptin | Risk of liver toxicity. | Do not prescribe or withdraw if there is liver failure or risk of liver failure; or if pre-treatment values of alanine aminotransferase (ALT) or aspartate aminotransferase (AST) > 3 times the higher limit of normal values. Evaluate risk factors. |  |
| **C-5** | *Reversible sodium-glucose cotransporter 2 inhibitors (SGLT2 inhibitors)*  (canagliflozin, dapagliflozin, empagliflozin) | Increased risk of limb amputation, urinary tract infections, falls, kidney damage, ketoacidosis, dehydration.  Has no efficacy when renal clearance is low. GFR <45ml/min/1.73m2, dapagliflozin <60 ml/min/1.73m2  Interaction with diuretics, laxatives: higher risk of orthostatic hypotension and dehydration.  Interaction with rifampicin, NSAIDs, contrast agents: higher risk of kidney damage.  Limited efficacy as a monotherapy. | Avoid if disease is being poorly controlled, complications associated with type-2 diabetes mellitus, dehydration risk. Evaluate history of amputations. Monitor fall risk and urinary tract infections.  Evaluate other, less toxic alternatives: DDP-4 inhibitors.  Avoid concomitant use. Exercise caution.  Treatment as a monotherapy: not recommended.  Evaluate alternatives of choice: metformin, glimepiride, gliclazide or DDP-4 inhibitors. | Wiviott et al, 2019 (20)  Ueda et al, 2018 (21)  Li et al, 2018 (22)  Neal et al, 2017 (23)  Lavalle et al, 2016 (24)  FDA, 2016 (25)  AEMPS, 2016 (26) |
| **C-6** | Glibenclamide and other long-acting sulfonylureas | Higher risk of prolonged hypoglycemia.  In glomerular filtration rate <30mL/min/1.73m2 (renal failure). | Contraindicated in elderly patients. Withdraw. Alternatives: metformin, glimepiride, gliclazide.  Withdraw in stages 4 and 5 of chronic kidney disease. Alternatives: Inhibitors of dipeptidyl peptidase 4. | O’Mahony et al, 2014 (Stopp) (27)  AGS Beers, 2019 (28)  Gangi et al, 2007 (29) |
| **C-7** | Metformin | Risk of weight loss.  Higher risk of lactic acidosis.  Suspend when the glomerular filtration rate <30mL/min/1.73m2 (renal failure). | Evaluate nutritional risk factors. Adjust dosing.  Evaluate risk factors (renal function).  Use with precaution in stage 3b. Avoid in stage IV or higher of chronic kidney disease. Prescribe other alternatives: DDP-4 inhibitors. | Inzucchi et al, 2014 (30) |
| **C-8** | Beta blockers | Risk of frequent episodes of hypoglycemia (1 or more episodes in one month).  Risk of severe hypotension, bradycardia, and heart block. | Evaluate the presence of episodes of hypoglycemia.  Avoid in type-2 diabetes mellitus.  Avoid in combination with non-dihydropyridine calcium channel blockers (verapamil, diltiazem).  Evaluate alternatives of choice: ACE inhibitors and/or ARBs, and/or calcium channel blockers. | JNC 8, 2014 (4)  O’Mahony et al, 2014 (Stopp) (27) |
|  | Non-selective | Risk of bronchospasm. | Do not use in patients with a history of bronchospasms. Evaluate alternatives. |  |
| **C-9** | Drugs that act on the Renin-angiotensin system (RAS). | Combination of RAS drugs: increased risk of hypopotassemia, hypotension and renal failure due to concomitant use (>1) of RAS drugs. | Withdraw ARB. The combination of ACE inhibitors + ARBs has been proven to not reduce cardiovascular or renal mortality, but it does increase the incidence of adverse drug reactions. | Makani et al, 2013 (32)  CADIME, 2017 (33)  AEMPS, 2017 (34) |
|  |  | Combination of RAS drug + diuretic + NSAID:  Increased risk of acute renal failure due to triple whammy. | Withdraw NSAID and substitute it for analgesics (acetaminophen). |  |
| **C-10** | Potassium-sparing diuretic (spironolactone, eplerenone) | Risk of severe hyperpotassemia >6.0 mEq/L when used in combination with RAS drugs. | Suspend potassium-sparing diuretic if it is being used in combination with RAS drugs. Otherwise, adjust dosing and monitor blood potassium levels at the beginning of the treatment, when dosage is increased, and regularly (every 6 months or at least once a year).  Appropriate starting dose of 12.5mg/day and adjust up to 25mg/day (spironolactone).  Evaluate other alternatives: Thiazide diuretics. | O’Mahony et al, 2014 (Stopp) (27)  AGS Beers, 2019 (28) |
| **C-11** | Proximal diuretics or loop diuretics. | Risk of hypopotassemia and hyperuricemia. | Adjust dosing and monitor blood potassium levels at the beginning of the treatment, when dosage is increased, and regularly (every 6 months or at least once a year). Monitor uric acid, evaluate risks in gout patients. | O’Mahony et al, 2014 (Stopp) (27) |
|  |  | Limited efficacy in isolated malleolar edema without clinical signs of heart failure. | Do not prescribe, withdraw. Use comprehensive measures and/or correct hypoalbuminemia. |  |
| **C-12** | Selective alpha-adrenergic agonist (alpha-methyldopa, clonidine) | Higher risk of sedation, dry mouth, hypotension, falling, bradycardia, and syncope. | It is not indicated as a first-line treatment for hypertension. Evaluate swallowing problems due to dry mouth. When suspended, rebound hypertension might appear.  Evaluate other alternatives: Thiazide diuretics and/or ACE inhibitors and/or ARBs, and/or calcium channel blockers. | Laroche et al, 2007 (35)  AGS Beers, 2019 (28) |
| **C-13** | Alpha blockers (prazosin, doxazosin) | Increased risk of orthostatic hypotension, bradycardia, recurrent syncope and falling. | It is not indicated as first-line treatment for hypertension. Do not use in heart failure. Evaluate falling risk.  Evaluate if treatment is for benign prostatic hyperplasia.  Evaluate other alternatives (if possible): Thiazide diuretics and/or ACE inhibitors and/or ARBs, and/or calcium channel blockers. | Laroche et al, 2007 (35)  AGS Beers, 2019 (28) |
| **C-14** | Nifedipine | Higher risk of orthostatic hypotension, stroke, falling, peripheral edema. | Avoid using as an antihypertensive drug, especially in conventional dosage forms (non-modified release).  Evaluate other alternatives (if possible): Thiazide diuretics and/or ACE inhibitors and/or ARBs, and/or other calcium channel blockers. | Laroche et al, 2007 (35) |
| **C-15** | Statins: High-intensity statin therapy Rosuvastatin 20mg-40mg Atorvastatin 80mg. | Higher risk of increased HbA1c, hyperglycemias, drug interactions and falling (risk of myopathy).  There is no evidence of efficacy when used in primary prevention. | Reduce high intensity therapy doses to low-moderate. Also, use low intensity therapy statins (simvastatin 10mg, pravastatin 10-20mg, lovastatin 20mg, fluvastatin 20-40mg, pitavastatin 1mg). Evaluate risk factors and interactions that might predispose myopathies.  There is no evidence of efficacy when used in primary prevention against cardiovascular diseases. | Cui et al, 2018 (6) Wilson et al, 1998 (REGICOR) (7)  Scott et al, 2009 (36)  AGS Beers, 2019 (28) |
| **C-16** | Fibrates (fenofibrate, ciprofibrate) | Low cardiovascular preventive efficacy in hypercholesterolemia.  Risk of cutaneous and hematologic adverse reactions, pain and muscle weakness. | If treatment with fibrates is considered, gemfibrozil is recommended.  Avoid concomitant use with statins. | Prescrire, 2015 (37).  Scott et al, 2009 (36) |
| **Other cardiovascular drugs C-17** | β-Methyl Digoxin | Increased risk of toxicity in doses higher than 0.125 μg/day or glomerular filtration rate (GFR) <30ml/min. | Avoid as a first line treatment for atrial fibrillation and heart failure. If necessary, adjust dosing according to renal function and monitor digoxin serum levels. | O’Mahony et al, 2014 (Stopp) (27)  AGS Beers, 2019 (28) |
| **C-18** | Anticoagulants | Risk of hemorrhage in patients with a low risk of thromboembolism^^[[1]](#footnote-0)^^.  Higher risk of bleeding when combined with other anticoagulants, antiplatelet drugs, NSAIDs, SSRIs, Ginkgo biloba. | Avoid use in patients with a low risk of thromboembolism (i.e.- Atrial fibrillation (AF) with CHA2DS2–VASc score=0, in male patients, - AF with CHA2DS2–VASc score =1, in female patients).  Avoid combinations. | Hansen et al, 2010 (38)  Pérez-Copete et al (39)  Stoddard et al (40) |
| **C-19** | Acetylsalicylic acid (ASA) | Unfavorable risk-benefit balance in primary prevention.  Increased risk of bleeding when using doses above 150mg/day.  Higher risk of bleeding when combined with other antiplatelet drugs. | Do not use.  Evaluate risk. Reduce dosage.  If dual antiplatelet therapy is in place for ischemic heart disease, withdraw one of the antiplatelet drugs (clopidogrel or other) if the length of treatment is ≥12 months.  If oral anticoagulants (coumarin drugs) are in place. Withdraw ASA. | O’Mahony et al, 2014 (Stopp) (27)  Butalia et al, 2011 (41)  Sutcliffe et al 2013 (42) |
| **COPD**  **C-20** | Theophylline | Low efficacy, higher risk of cardiac toxicity and interactions. | Do not use. Evaluate risk factors.  Alternative: Inhaled bronchodilators. | O’Mahony et al, 2014 (Stopp) (27) |
| **C-21** | Inhaled Corticosteroids | Low efficacy as a monotherapy. | Do not use as a monotherapy. Evaluate mixed disease typologies (asthma/COPD).  Recommended drug combinations: formoterol/beclomethasone, formoterol/budesonide, salmeterol/fluticasone, formoterol/fluticasone | Tashkin & Strange, 2018 (43)  ICS, 2018 (44) |
| **C-22** | Proton-pump inhibitors (omeprazole, lansoprazole, esomeprazole, pantoprazole, rabeprazole) | Increased risk of osteoporotic fractures, pneumonia, *C. difficile* infections, diarrhea, hypomagnesemia, vitamin B12 deficiency, and interactions. | Evaluate length of treatment and indication. Avoid use for >8 weeks unless there is a need to continue the treatment. When considering if treatment with PPIs should be reinstated after a treatment that lasted 8 or less weeks, evaluate if there is a need to restart treatment of if H2 blocker therapy failed. Evaluate drug-drug interactions. | O’Mahony et al, 2014 (Stopp) (27)  AGS Beers, 2019 (28) |
| **C-23** | Tricyclic antidepressants (amitriptyline, imipramine, doxepin) | Risk of serotonin syndrome and anticholinergic effects.  Risk of interactions and falling when used together with benzodiazepines. | Do not use in minor depressive disorder, reactive depression. Evaluate risk factors in older adult patients (risk of delirium, falling, dry mouth, constipation, history of cognitive disorders), consider non-pharmacological measures.  Evaluate risk factors for falling risk. Avoid concomitant use with other tricyclic antidepressants.  Alternatives: SSRIs (i.e. sertraline, fluoxetine), do not associate with MAOIs. Use low doses and for the least amount of time possible. | Cortajarena et al, 2016 (45)  Fuentes & Mérida, 2011 (8) |
| **C-24** | Long-acting benzodiazepines (diazepam, clonazepam, clobazam) | Risk of excessive sedation, confusion, respiratory depression, gait instability, falling, bone fractures. | Withdraw. Do not use as a hypnotic and/or anxiolytic drug. Follow non-pharmacological treatments for insomnia.  Alternatives: i.e. Lorazepam, alprazolam, Z-drugs (zolpidem, zopiclone). Use the lowest dose possible, for the shortest period of time possible (no more than 4 weeks, including tapering period). If the health condition persists, reconsider the therapeutic approach. | CADIME, 2017 (33)  AGS Beers, 2019 (28) |
| **C-25** | Psycholeptic drugs (anxiolytics, antipsychotics, psychoactives, hypnotics, sedatives and antidepressants) | Higher risk of gait abnormalities, parkinsonism, sedation, constipation, urinary retention, drug-drug interactions with other sedative drugs (including opioids); falling risk and bone fractures.  Higher risk of increased levels of HbA1c with clozapine and olanzapine. Risk of patient developing type-2 diabetes mellitus. | Evaluate risk factors (history of falling, or risk of falling). Avoid using more than 3 psychotropic drugs as efficacy is not increased, but adverse events are.  Be especially cautious with patients suffering from parkinsonism, Lewy body dementia and diabetes mellitus.  Avoid off-label uses.  Use of atypical antipsychotics (clozapine, olanzapine) is not recommended. | Berdot et al, 2009 (46)  O’Mahony et al, 2014 (Stopp) (27)  Proietto, 2004 (47) |
| **C-26** | Anticholinergics (antidepressants, antihistamines, urinary anticholinergics) | Increased risk of worsening closed angle glaucoma^^[[2]](#footnote-1)^^. | Do not use. Follow recommendations stated in other sections. | Laroche et al, 2007 (35) |
| **Drugs for neurocognitive disorders**  **C-27** | Acetylcholinesterase inhibitors (donepezil, galantamine, rivastigmine) | Absence of response to treatment after 12 weeks.  When they are not indicated^^[[3]](#footnote-2)^^, worsening of patient status (score ≥6 in global deterioration scale and functional assessment staging test (FAST) ≥ stage 6). | If there is no response to treatment after 12 weeks, withdraw after tapering.  Discontinue usage.  Alternatives: provide more and better care by caregivers and family members. | Prescrire, 2017 (48).  AGS Beers, 2019 (28) |
| **Pain**  **C-28** | Opioids | Risk of dependence, tolerance, sedation, dizziness, vertigo and somnolence.  Higher risk of respiratory depression, urinary retention, constipation, falling, cognitive decline (in neurocognitive disorders). | Avoid in patients with a high risk of falling (≥ 1 fall in the last three months). Evaluate therapeutic objective. Prescribe according to pain scale and progression time. Adjust dosing based on pain scale and progression time. Do not prescribe meperidine.  Alternative: acetaminophen (paracetamol). | O’Mahony et al, 2014 (Stopp) (27)  AGS Beers, 2019 (28)  Holt, 2010 (49)  Renom-Guiteras 2015 (50)  Seppala et al, 2018 (51) |
| **C-29** | NSAIDs (Cox 1 and Cox 2)  (Cox2) | Higher risk of gastrointestinal, renal and cardiovascular adverse effects.  Risk of bleeding reappearance.  Higher risk of cardiovascular disorders. | Evaluate therapeutic objective. If necessary, prescribe for the shortest time possible alongside gastroprotection. Reevaluate indication.    Evaluate risks when there is a history of peptic ulcer or digestive hemorrhage.  Evaluate risks in patients with a history of cardiovascular diseases (HTN, MI, HF, etc.)  Consider intermittent use. | Laroche et al, 2007 (35)  Aranguren, 2016 (Bit Navarra) (52) |
| **Miscellaneous**  **C-30** | First generation H_1_-antihistamines. | When used for longer than 1 week, it may cause pronounced anticholinergic effects (incontinence, constipation, sedation, falling risk). | Avoid use. If necessary, withdraw after first week of treatment. | O’Mahony et al, 2014 (Stopp) (27)  AGS Beers, 2019 (28) |
| **C-31** | Slow-acting drugs for osteoarthritis treatment (diacerein, glucosamine, chondroitin) | Questionable efficacy against osteoarthritis (marginal therapeutic value), risk of hepatic and gastrointestinal disorders, skin reactions. | Avoid use. | O’Mahony et al, 2014 (Stopp) (27) |
| **C-32** | Urinary antispasmodics (oxybutynin, tolterodine, darifenacin) | Limited efficacy against urinary incontinence.  Risk of increased confusion and agitation in patients with dementia or chronic cognitive impairment. Risk of acute exacerbation of glaucoma in patients with closed-angle glaucoma. Risk of urinary retention in patients with chronic prostatism. | Avoid use.  If necessary, reevaluate the need to continue treatment. | O’Mahony et al, 2014 (Stopp) (27) |
| **C-33** | Bisphosphonates | Very limited efficacy in primary prevention of bone fracture incidence (risk of esophagitis, atypical bone fractures, osteonecrosis of the jaw). | Prescribe according to FRAX score in order to determine if use in primary prevention is appropriate. If necessary, treatment should not exceed 5 years.  Evaluate falling risk (avoid falls). | Anagnostis & Stevenson, 2015 (53)  Butlletì groc – FICF 2009 (54)  Wells et al, 2008 (55). |

T2DM: Type-2 diabetes mellitus. CNS: nervous central system. BMI: body mass index. NPH: Neutral Protamine Hagedorn. DDP-4 inhibitors: Dipeptidyl peptidase-4 inhibitors. GFR: glomerular filtration rate. HTN: hypertension. MI: myocardial infarction. HF: heart failure. NSAIDs: nonsteroidal anti-inflammatory drugs. SSRI: selective serotonin reuptake inhibitors. HbA1c: glycated hemoglobin. MAOIs: Monoamine oxidase inhibitors. COPD: Chronic obstructive pulmonary disease. ACE inhibitors: Angiotensin-converting-enzyme inhibitors. ARB: Angiotensin II receptor blockers. RAS: Renin-angiotensin system. ASA: Acetylsalicylic acid. PPIs: Proton-pump inhibitors

**REFERENCES**

1. Farrell B, Black C, Thompson W, McCarthy L, Rojas-Fernandez C, Lochnan H, et al. Deprescribing antihyperglycemic agents in older persons. Canadian Family Physician. 2017;63(11):832.

2. Qaseem A, Wilt TJ, Kansagara D, et al. Hemoglobin a1c targets for glycemic control with pharmacologic therapy for nonpregnant adults with type 2 diabetes mellitus: A guidance statement update from the american college of physicians. Annals of Internal Medicine. 2018;168(8):569-76.

3. ICS. Argumentario de antidiabéticos no insulínicos. Bases científicas utilizadas para elaborar el EQPF en medicina familiar y comunitaria. Cataluña: Instituto Catalán de la Salud; 2018.

4. James PA, Oparil S, Carter BL, et al. 2014 evidence-based guideline for the management of high blood pressure in adults: Report from the panel members appointed to the eighth joint national committee (jnc 8). JAMA. 2014;311(5):507-20.

5. Solini A, Grossman E. What Should Be the Target Blood Pressure in Elderly Patients With Diabetes? Diabetes Care. 2016;39 Suppl 2:S234-43.

6. Cui JY, Zhou RR, Han S, Wang TS, Wang LQ, Xie XH. Statin therapy on glycemic control in type 2 diabetic patients: A network meta-analysis. J Clin Pharm Ther. 2018.

7. Wilson PWF, D’Agostino RB, Levy D, Belanger AM, Silbershatz H, Kannel WB. Prediction of Coronary Heart Disease Using Risk Factor Categories. Circulation. 1998;97(18):1837.

8. Fuentes Cuenca S, Mérida Casado E. Protocolo terapéutico de la depresión en el anciano. 2011;10(86):5851-4.

9. AEMPS. Informe de Posicionamiento Terapéutico de insulina degludec/liraglutida (Xultophy®) en diabetes mellitus tipo 2. España: Agencia española de medicamentos y productos sanitarios; 2018. p. 1-7.

10. Rosenstock J, Cheng A, Ritzel R, Bosnyak Z, Devisme C, Cali AMG, et al. More Similarities Than Differences Testing Insulin Glargine 300 Units/mL Versus Insulin Degludec 100 Units/mL in Insulin-Naive Type 2 Diabetes: The Randomized Head-to-Head BRIGHT Trial. Diabetes Care. 2018;41(10):2147-54.

11. Rys P, Wojciechowski P, Rogoz-Sitek A, Niesyczyński G, Lis J, Syta A, et al. Systematic review and meta-analysis of randomized clinical trials comparing efficacy and safety outcomes of insulin glargine with NPH insulin, premixed insulin preparations or with insulin detemir in type 2 diabetes mellitus. Acta diabetologica. 2015;52(4):649-62.

12. Canadian Agency for Drugs and Technologies in H. Long-Acting Insulin Analogues for the Treatment of Diabetes Mellitus: Meta-analyses of Clinical Outcomes. CADTH technology overviews. 2010;1(1):e0113-e.

13. Basal Insulin and Cardiovascular and Other Outcomes in Dysglycemia. New England Journal of Medicine. 2012;367(4):319-28.

14. AEMPS. Informe de Posicionamiento Terapéutico de dulaglutida (Trulicity®) España: Agencia Española de Medicamentos y Productos Sanitarios; 2016 [Available from: http://www.aemps.gob.es/medicamentosUsoHumano/informesPublicos/docs/IPT-dulaglutida-trulicity.pdf.

15. Marso SP, Daniels GH, Brown-Frandsen K, Kristensen P, Mann JFE, Nauck MA, et al. Liraglutide and Cardiovascular Outcomes in Type 2 Diabetes. New England Journal of Medicine. 2016;375(4):311-22.

16. FDA. FDA Drug Safety Communication: FDA adds warnings about heart failure risk to labels of type 2 diabetes medicines containing saxagliptin and alogliptin USA: Food and Drug Administration; 2014 [Available from: https://www.fda.gov/downloads/Drugs/DrugSafety/UCM493965.pdf.

17. White WB, Bakris GL, Bergenstal RM, Cannon CP, Cushman WC, Fleck P, et al. EXamination of cArdiovascular outcoMes with alogliptIN versus standard of carE in patients with type 2 diabetes mellitus and acute coronary syndrome (EXAMINE): a cardiovascular safety study of the dipeptidyl peptidase 4 inhibitor alogliptin in patients with type 2 diabetes with acute coronary syndrome. Am Heart J. 2011;162(4):620-6.e1.

18. Rosenstock J, Perkovic V, Johansen OE, Cooper ME, Kahn SE, Marx N, et al. Effect of Linagliptin vs Placebo on Major Cardiovascular Events in Adults With Type 2 Diabetes and High Cardiovascular and Renal Risk: The CARMELINA Randomized Clinical Trial. Jama. 2019;321(1):69-79.

19. Abrahami D, Douros A, Yin H, Yu OHY, Renoux C, Bitton A, et al. Dipeptidyl peptidase-4 inhibitors and incidence of inflammatory bowel disease among patients with type 2 diabetes: population based cohort study. BMJ. 2018;360.

20. Wiviott SD, Raz I, Bonaca MP, Mosenzon O, Kato ET, Cahn A, et al. Dapagliflozin and Cardiovascular Outcomes in Type 2 Diabetes. N Engl J Med. 2019;380(4):347-57.

21. Ueda P, Svanstrom H, Melbye M, Eliasson B, Svensson AM, Franzen S, et al. Sodium glucose cotransporter 2 inhibitors and risk of serious adverse events: nationwide register based cohort study. Bmj. 2018;363:k4365.

22. Li D, Yang JY, Wang T, Shen S, Tang H. Risks of diabetic foot syndrome and amputation associated with sodium glucose co-transporter 2 inhibitors: A Meta-analysis of Randomized Controlled Trials. Diabetes Metab. 2018;44(5):410-4.

23. Neal B, Perkovic V, Mahaffey KW, de Zeeuw D, Fulcher G, Erondu N, et al. Canagliflozin and Cardiovascular and Renal Events in Type 2 Diabetes. New England Journal of Medicine. 2017;377(7):644-57.

24. Lavalle-González F, Eliaschewitz F, Cerdas S, Chacon MP, Tong C, Alba M. Efficacy and safety of canagliflozin in patients with type 2 diabetes mellitus from Latin America. Current medical research and opinion [Internet]. 2016; 32(3):[427-39 pp.]. Available from: http://onlinelibrary.wiley.com/o/cochrane/clcentral/articles/615/CN-01200615/frame.html.

25. FDA. FDA Drug Safety Communication: FDA strengthens kidney warnings for diabetes medicines canagliflozin (Invokana, Invokamet) and dapagliflozin (Farxiga, Xigduo XR) USA: Food and Drug Administration; 2016 [Available from: https://www.fda.gov/Drugs/DrugSafety/ucm505860.htm.

26. AEMPS. Informe de Posicionamiento Terapéutico de canagliflozina (Invokana®) España: Agencia española de medicamentos y productos sanitarios; 2016 [Available from: http://www.aemps.gob.es/medicamentosUsoHumano/informesPublicos/docs/IPT-canagliflozina-invokana.pdf.

27. O'Mahony D, O'Sullivan D, Byrne S, O'Connor MN, Ryan C, Gallagher P. STOPP/START criteria for potentially inappropriate prescribing in older people: version 2. Age and Ageing. 2014.

28. AGS. American Geriatrics Society 2019 Updated AGS Beers Criteria(R) for Potentially Inappropriate Medication Use in Older Adults. J Am Geriatr Soc. 2019;67(4):674-94.

29. Gangji AS, Cukierman T, Gerstein HC, Goldsmith CH, Clase CM. A systematic review and meta-analysis of hypoglycemia and cardiovascular events: a comparison of glyburide with other secretagogues and with insulin. Diabetes Care. 2007;30(2):389-94.

30. Inzucchi SE, Lipska KJ, Mayo H, Bailey CJ, McGuire DK. Metformin in patients with type 2 diabetes and kidney disease: a systematic review. Jama. 2014;312(24):2668-75.

31. Huang Y, Lai C, Wang Y, Wang C, Wang J, Wang H, et al. Impact of selective and nonselective beta-blockers on the risk of severe exacerbations in patients with COPD. International Journal of Chronic Obstructive Pulmonary Disease. 2017;12:2987-96.

32. Makani H, Bangalore S, Desouza KA, Shah A, Messerli FH. Efficacy and safety of dual blockade of the renin-angiotensin system: meta-analysis of randomised trials. BMJ : British Medical Journal. 2013;346.

33. CADIME. Revisión de prescripciones para evitar problemas de seguridad Andalucía: Boletín terapéutico andaluz. Centro andaluz de documentación e información de medicamentos; 2017 [Available from: http://www.cadime.es/docs/bta/CADIME_BTA_32_1.pdf.

34. AEMPS. Uso combinado de medicamentos que actúan sobre el sistema renina-angiotensina (IECA/ARA II): restricciones de uso España: Agencia española de medicamentos y productos sanitarios; 2014 [Available from: https://www.aemps.gob.es/informa/notasInformativas/medicamentosUsoHumano/seguridad/2014/NI-MUH_FV_06-renina-angiotensina.htm.

35. Laroche ML, Charmes JP, Merle L. Potentially inappropriate medications in the elderly: a French consensus panel list. Eur J Clin Pharmacol. 2007;63(8):725-31.

36. Scott D, Blizzard L, Fell J, Jones G. Statin therapy, muscle function and falls risk in community-dwelling older adults. Qjm. 2009;102(9):625-33.

37. PRESCRIRE. Pour mieux soigner, des médicaments à écarter: bilan 2015. Rev Prescrire. 2015;35(376):144-51.

38. Hansen ML, Sorensen R, Clausen MT, Fog-Petersen ML, Raunso J, Gadsboll N, et al. Risk of bleeding with single, dual, or triple therapy with warfarin, aspirin, and clopidogrel in patients with atrial fibrillation. Arch Intern Med. 2010;170(16):1433-41.

39. Pérez-Copete J, Esteve-Pastor MA, Roldán V, Valdés M, Marín F. Escalas de evaluación del riesgo tromboembólico y hemorrágico en la fibrilación auricular. Revista Española de Cardiología. 2016;16(Supl.A):25-32.

40. Stoddard GJ, Archer M, Shane-McWhorter L, Bray BE, Redd DF, Proulx J, et al. Ginkgo and Warfarin Interaction in a Large Veterans Administration Population. AMIA Annu Symp Proc. 2015;2015:1174-83.

41. Butalia S, Leung AA, Ghali WA, Rabi DM. Aspirin effect on the incidence of major adverse cardiovascular events in patients with diabetes mellitus: a systematic review and meta-analysis. Cardiovasc Diabetol. 2011;10:25.

42. Sutcliffe P, Connock M, Gurung T, Freeman K, Johnson S, Ngianga-Bakwin K, et al. Aspirin in Primary Prevention of Cardiovascular Disease and Cancer: A Systematic Review of the Balance of Evidence from Reviews of Randomized Trials. PLOS ONE. 2013;8(12):e81970.

43. Tashkin DP, Strange C. Inhaled corticosteroids for chronic obstructive pulmonary disease: what is their role in therapy? International journal of chronic obstructive pulmonary disease. 2018;13:2587-601.

44. ICS. Estandar de calidad de la prescripción farmacéutica. Cataluña: Instituto Catalán de Salud; 2018.

45. Cortajarena García MC, Ron Martin S, Miranda Vicario E, Ruiz de Vergara Eguino A, Azpiazu Gomez PJ, Lopez Aldana J. Antidepresivos en la tercera edad. Medicina de Familia SEMERGEN. 2016;42(7):458-63.

46. Berdot S, Bertrand M, Dartigues J-F, Fourrier A, Tavernier B, Ritchie K, et al. Inappropriate medication use and risk of falls – A prospective study in a large community-dwelling elderly cohort. BMC Geriatrics. 2009;9:30-.

47. Proietto J. Diabetes and Antipsychotic Drugs. Australian Prescriber 2004;27(5):118-9.

48. PRESCRIRE. Pour mieux soigner, des médicaments à écarter: bilan 2017. Rev Prescrire. 2017;37(400):1374-148.

49. Holt S, Schmiedl S, Thürmann PA. Potentially Inappropriate Medications in the Elderly: The PRISCUS List. Deutsches Ärzteblatt International. 2010;107(31-32):543-51.

50. Renom-Guiteras A, Meyer G, Thurmann PA. The EU(7)-PIM list: a list of potentially inappropriate medications for older people consented by experts from seven European countries. Eur J Clin Pharmacol. 2015;71(7):861-75.

51. Seppala LJ, van de Glind EMM, Daams JG, Ploegmakers KJ, de Vries M, Wermelink A, et al. Fall-Risk-Increasing Drugs: A Systematic Review and Meta-analysis: III. Others. J Am Med Dir Assoc. 2018;19(4):372.e1-.e8.

52. Aranguren Ruiz I, Elizondo Rivas G, Azparren Andia A. Consideraciones de seguridad de los AINE. 2016;24(2):1-13.

53. Anagnostis P, Stevenson JC. Bisphosphonate drug holidays--when, why and for how long? Climacteric. 2015;18 Suppl 2:32-8.

54. FICF. Bifosfonatos: una relación beneficio-riesgo dudosa. Butlletí Groc. 2009;22(3):9-12.

55. Wells GA, Cranney A, Peterson J, Boucher M, Shea B, Robinson V, et al. Alendronate for the primary and secondary prevention of osteoporotic fractures in postmenopausal women. Cochrane Database Syst Rev. 2008(1):Cd001155.

1. Evaluate bleeding risk with HAS-BLED score. [↑](#footnote-ref-0)
2. Consider the anticholinergic effects in the worsening condition of other pathologies like prostatic hyperplasia, dementia, risk of delirium. [↑](#footnote-ref-1)
3. Applies to certain types of neurodegenerative dementias like Alzheimer’s disease, and other cognitive impairment etiologies (vascular dementia, prion dementia, among others). [↑](#footnote-ref-2)
