# Supplementary material for: Tackling potentially inappropriate prescriptions in older adults: development of deprescribing criteria by consensus from experts in Colombia, Argentina, and Spain
Source: BMC Geriatr. 2023 Oct 20;23:682. doi: 10.1186/s12877-023-04271-9 (PMC10588094; doi:10.1186/s12877-023-04271-9)
Supplement: Supplementary file 2 — Additional file 2. Sociodemographic and clinical characteristics of the cohort (n=36111). [file 12877_2023_4271_MOESM2_ESM.docx]

Additional file 2. Sociodemographic and clinical characteristics of the cohort. (n=36111)

| **Sex/Age (Years)** | **60-74** | **75-90** | **>90** | **N (%)** |
| --- | --- | --- | --- | --- |
| Male | 12951 | 6317 | 322 | 19590 (54.25) |
| Female | 10388 | 5785 | 348 | 16521 (45.75) |
| **Pluripathology (number of diseases)**  OR=1.39 (IC95% 1.33-1.46) P <0.0001 | **60-74** | **75-90** | **>90** | **N (%)** |
| <3 | 9054 | 3796 | 190 | 13040 (36.11) |
| ≥3 | 14285 | 8306 | 480 | 23071(63.89) |
| **Number of drugs**  OR=1.43 (IC95% 1.36-1.51) P <0.0001 | **60-74** | **75-90** | **>90** | **N (%)** |
| <5 | 5786 | 2263 | 123 | 8172 (22.63) |
| 5 y 10 | 12258 | 5940 | 331 | 18529 (51.31) |
| 11 y 20 | 4963 | 3660 | 200 | 8823 (24.43) |
| >20 | 332 | 239 | 16 | 587 (1.63) |
| **Diagnoses International Classification of Diseases (ICD-10)** | **n** | | **%** | |
| **Diseases of the circulatory system** |  | |  | |
| Hypertensive diseases | 19223 | | 53.23 | |
| Other and unspecified disorders of the circulatory system  (pericarditis, endocarditis, trastornos valvulares y otros) | 1488 | | 4.12 | |
| **Endocrine, nutritional and metabolic diseases** |  | |  | |
| Disorders of thyroid gland | 1945 | | 5.38 | |
| Metabolic disorders | 1446 | | 4.00 | |
| **Diseases of the nervous system** |  | |  | |
| Polyneuropathies and other disorders of the peripheral nervous system | 892 | | 2.47 | |
| **Diseases of the respiratory system** |  | |  | |
| Chronic lower respiratory diseases | 469 | | 1.30 | |
| **Diseases of the digestive system** |  | |  | |
| Diseases of esophagus, stomach and duodenum | 836 | | 2.31 | |
| **Diseases of the musculoskeletal system and connective tissue** |  | |  | |
| Osteoarthritis | 1784 | | 4.94 | |
| **Diseases of the genitourinary system** |  | |  | |
| Acute kidney failure and chronic kidney disease | 1561 | | 4.32 | |
| International Classification of Diseases. | | | | |

Source: Self-elaboration.
