# Supplementary material for: Tackling potentially inappropriate prescriptions in older adults: development of deprescribing criteria by consensus from experts in Colombia, Argentina, and Spain
Source: BMC Geriatr. 2023 Oct 20;23:682. doi: 10.1186/s12877-023-04271-9 (PMC10588094; doi:10.1186/s12877-023-04271-9)
Supplement: Supplementary file 3 — Additional file 3. Pharmacological groups prescribed. [file 12877_2023_4271_MOESM3_ESM.docx]

Additional file 3. Pharmacological groups prescribed.

| **ATC code** | **Level** | **N** | **%** |
| --- | --- | --- | --- |
| A10 | Drugs used in Diabetes | 32246 | 89.30 |
| *A10BA02* | *Metformin* | *15600* | *48.38%* |
| *A10BB* | *Sulfonylureas* | *4095* | *12.70%* |
| *A10AB* | *Insulins and analogues for injection, fast-acting* | *2234* | *6.93%* |
| *A10AE* | *Insulins and analogues for injection, long-acting* | *6278* | *19.47%* |
| C09 | Agents acting on the renin-angiotensin system | 24143 | 66.86 |
| C10 | Lipid modifying agents | 24115 | 66.78 |
| B01 | Antithrombotic agents | 20203 | 55.95 |
| N02 | Analgesics | 14073 | 38.97 |
| A02 | Drugs for acid related disorders | 13615 | 37.70 |
| C03 | Diuretics | 10804 | 29.92 |
| C08 | Calcium channel blockers | 10481 | 29.02 |
| C07 | Beta blocking agents | 9330 | 25.84 |
| H03 | Thyroid therapy | 7172 | 19.86 |
| A11 | Vitamins | 6710 | 18.58 |
| J01 | Antibacterials for systemic use | 6626 | 18.35 |
| S01 | Ophthalmologicals | 4948 | 13.70 |
| N06 | Psychoanaleptics | 4557 | 12.62 |
| M01 | Antiinflammatory and antirheumatic products | 4145 | 11.48 |

**ATC: Anatomical, Therapeutic, Chemical classification system.**

Source: Self-elaboration.
