# Supplementary material for: Tackling potentially inappropriate prescriptions in older adults: development of deprescribing criteria by consensus from experts in Colombia, Argentina, and Spain
Source: BMC Geriatr. 2023 Oct 20;23:682. doi: 10.1186/s12877-023-04271-9 (PMC10588094; doi:10.1186/s12877-023-04271-9)
Supplement: Supplementary file 4 — Additional file 4. Validity and Reliability of some criteria. [file 12877_2023_4271_MOESM4_ESM.docx]

Additional file 4. Validity and Reliability of some criteria..

|  | **Essential** | **Useful but not essential** | **Not necessary** | **CVR** | **CVR'** | **Kappa** | **Strength of agreement*** |
| --- | --- | --- | --- | --- | --- | --- | --- |
| **R1. (T2DM)** | 17 | 1 | 0 | 0.88 | 0.94 | 0.83 | Almost perfect |
| **R2. (T2DM)** | 18 | 0 | 0 | 1.00 | 1.00 | 1.00 | Almost perfect |
| **R3. Hipertensión arterial** | 16 | 2 | 0 | 0.77 | 0.88 | 0.69 | Substantial |
| **R4. Dyslipidemia** | 15 | 2 | 1 | 0.66 | 0.83 | 0.54 | Moderate |
| **R5. CNS disorders** | 15 | 3 | 0 | 0.66 | 0.83 | 0.56 | Moderate |
| **C1.Insulin degludec (Alone and in combination with liraglutide)** | 16 | 2 | 0 | 0.77 | 0.88 | 0.69 | Substantial |
| **C2. Insulin glargine** | 16 | 2 | 0 | 0.77 | 0.88 | 0.69 | Substantial |
| **C3. GLP1** | 16 | 2 | 0 | 0.77 | 0.88 | 0.69 | Substantial |
| **C4. DPP-4 inhibitors** | 16 | 2 | 0 | 0.77 | 0.88 | 0.69 | Substantial |
| **C5. SGLT2 inhibitors** | 16 | 2 | 0 | 0.77 | 0.88 | 0.69 | Substantial |
| **C6. Glibenclamide and other long-acting sulfonylureas** | 18 | 0 | 0 | 1.00 | 1.00 | 1.00 | Almost perfect |
| **C7. Metformin** | 18 | 0 | 0 | 1.00 | 1.00 | 1.00 | Almost perfect |
| **C8. Beta blockers** | 16 | 2 | 0 | 0.77 | 0.88 | 0.69 | Substantial |
| **C9.Drugs that act on the Renin-angiotensin system (RAS)** | 15 | 3 | 0 | 0.66 | 0.83 | 0.56 | Moderate |
| **C21. Inhaled Corticosteroids** | 15 | 3 | 0 | 0.66 | 0.83 | 0.56 | Moderate |
| **C34. Laxatives** | 7 | 11 | 0 | -0.22 | 0.38 | 0.25 | Fair |
| **Total** | **613** | **86** | **3** | **29** | **34** | **0.67 (95% CI**  **0.63-0.72)** | **Substantial** |
| **S-CVI** |  |  |  | **0.74** | **0.87** |  |  |
| **S-CVI (accepted items)** |  |  |  | **0.75** | **0.87** |  |  |
| **Fleiss’ Kappa (accepted items)** |  |  |  |  |  | **K 0.69 (95% CI**  **0.64-0.73)** | **Substantial** |

* Criteria for the Interpretation of Kappa values by Landis & Koch.

K: Fleiss’ Kappa. CVR: content validity ratio. CVR’: adjustment proposed by Tristan. S-CVI: Scale-level Content Validity Index.

T2DM: Type-2 diabetes mellitus. GLP-1: Glucagon-like peptide 1 receptor agonists). DDP-4: dipeptidyl peptidase 4 inhibitors. SGLT2 inhibitors: Reversible sodium-glucose cotransporter 2 inhibitors.
